# Neurocognitive Profiles in Parents of Autistic Children and Parents of Children with Anorexia Nervosa

**DOI:** 10.1007/s10803-025-06871-3

**Published:** 2025-05-15

**Authors:** Rosemary Neville-Jones, Tracy Garvin, Peter G. Enticott, Matthew Fuller-Tyszkiewicz, Melissa Kirkovski

**Affiliations:** School of Psychology, Deakin University, Burwood, Australia

**Keywords:** Anorexia nervosa, Autism spectrum disorder, Heritability, Executive function, Cognitive flexibility, Mentalizing

## Abstract

There is growing evidence to suggest an overlap between autism spectrum disorder (autism) and anorexia nervosa (AN), both of which are known to be highly heritable conditions. The aim of this study was to explore overlapping behavioural and clinical characteristics, and neurocognitive profiles related to the autism dyad of symptoms among *parents*. Parents of autistic children, parents of children with AN, and parents of typically developing children completed a battery of behavioural questionnaires and neurocognitive tasks related to social cognition and cognitive flexibility. Parents of autistic children reported significantly more difficulties on imaginative abilities compared to the other two parent groups. Parents of children with AN had superior performance on cognitive flexibility tasks. As expected, and in support of an overlap between the two conditions, increased eating disorder psychopathology was associated with poorer performance on neurocognitive tasks related to the autism dyad. Understanding the overlap between AN and autism has important implications for accurate development of risk profiles, diagnosis, as well as treatment. This is critical as those with overlapping traits of autism and AN have poorer treatment outcomes and are at higher risk of the damaging physical health consequences of AN.

**Supplementary Information:**

The online version contains supplementary material available at https://doi.org/10.1007/s10803-025-06871-3.

Research increasingly suggests an overlap between two seemingly disparate conditions; autism spectrum disorder (autism) and anorexia nervosa (AN) (Baron-Cohen et al., 2013; Boltri & Sapuppo, 2021; Brown & Stokes, 2020; Fithall et al., 2023; Gillberg, 1985; Huke et al., 2013; Oldershaw et al., 2011; Susanin et al., 2022; Tchanturia et al., 2013; Westwood & Tchanturia, 2017). Autism is a neurodevelopmental disorder diagnosed behaviourally by a dyad of symptoms comprising persistent deficits in *social communication and interaction*, and *restricted and repetitive patterns of behaviour*,* interests*,* and activities* (RRBI; (APA, 2022). In contrast, AN is an eating disorder characterised by low body weight, a strict restriction of food or calories, excessive exercise, and an intense fear of gaining weight (APA, 2022). Interest in the overlap between these disorders is underscored by significant psychiatric comorbidity between the two conditions (Nickel et al., 2019) and similarities in aspects of their behavioural presentation. Specifically, behaviours reflecting the autism dyad, including rigidity in thinking and behaviour, and difficulties in social domains, are often observed in individuals with AN (Boltri & Sapuppo, 2021; Inal-Kaleli et al., 2025; Oldershaw et al., 2011). Indeed, recent evidence identified the presence of clinically significant levels of autistic traits in up to 30% of individuals with AN (Brown & Stokes, 2020).

Understanding the factors contributing to the overlap observed between autism and AN has important clinical implications. AN has a standardised mortality rate of up to 15.9%, among the highest across psychiatric disorders (Guinhut et al., 2021). AN is particularly difficult to treat (Fairburn, 2005), and troublingly, when high levels of autistic traits are present in individuals with AN, they are shown to experience significantly worse clinical outcomes, such as poor response to treatment (Nielsen et al., 2015; Tchanturia et al., 2016), and detrimental physiological health outcomes such as cardiac and gastrointestinal problems (Cass et al., 2020). Better understanding the role of autistic traits in AN and the overlap between the two disorders is crucial for improved clinical outcomes.

Despite growing evidence in this area, it is not clear why this overlap between AN and autism exists. One theory suggests that biological sex differences may impact the presentation of behavioural symptoms, leading to misdiagnosis. For example, in regards to the RRBI aspect of the autism dyad, females may be more likely to fixate on eating, body image or exercise, rather than interests typically observed among males (such as in trains and mechanics), which consequently may be mistakenly diagnosed as an eating disorder (Hull et al., 2020; Kirkovski et al., 2013). This pattern of behaviour is distinct from disordered feeding and eating frequently observed in autism due to sensory preferences, as it has a specific focus on weight and body image (Baraskewich et al., 2021). Another idea is that issues related to the dyad, including social difficulties and inflexible thinking, are observed in AN populations as a consequence of malnutrition experienced during acute phases of the illness (Gray et al., 2024; Hirst et al., 2017). Support for the ‘starvation theory’ is grounded in seminal research by Keys et al. (1950) which found significant cognitive changes, including increased preoccupation with food and social problems among young males following a prolonged period of starvation. Some researchers therefore suggest that starvation and associated neurobiological changes can account for some cognitive and behavioural difficulties among AN patients (van Noort et al., 2016). However, challenges with this idea include evidence that difficulties persist among individuals who have recovered from the acute phase of the illness (Weinbach et al., 2019) as well as retrospective reports of social difficulties in childhood prior to the onset of the illness (Krug et al., 2013), indicating there may be an underlying predisposition to these features.

Accordingly, heritable factors have also been theorised to contribute to the overlap between AN and autism. It is possible that individuals with AN may have an underlying genetic vulnerability, and this vulnerability is shared with autistic individuals, contributing to the intersection between the two disorders. Genetic links are evident in both conditions; a meta-analysis of twin studies estimated that heritability rates for autism are between 64% and 91% (Tick et al., 2016). In AN, family studies demonstrated that first-degree relatives of individuals diagnosed with AN have a tenfold greater risk of developing AN themselves (Bulik et al., 2007). In support for an overlapping vulnerability, AN is more likely to be present in individuals with a family history of autism (Rastam, 2007), and autism is evident in families with a history of AN (Koch et al., 2015). Research also shows that individuals with a sibling diagnosed with a neurodevelopmental disorder (autism or attention deficit hyperactivity disorder; ADHD) are at higher risk of developing any feeding and eating disorder (Christiansen et al., 2024). Of specific relevance to this project, individuals with AN were also reported to have had higher autistic polygenic risk scores compared to controls. Christiansen et al. (2024) also report that those with a maternal half-sibling with autism were at greater risk of AN. An increased risk of eating disorder psychopathology (eating disorder not otherwise specified) was also noted for individuals who had a cousin with autism, but not those who had a cousin with ADHD (Christiansen et al., 2024). This emphasises the likelihood of a biological mechanism contributing to the overlapping presentation of autism and AN. Further, sub-clinical difficulties related to the autism dyad have been observed in family members of individuals with AN (Galimberti et al., 2013; Kanakam et al., 2013; Lang et al., 2016). It is, therefore, possible that individuals with autism and AN have a shared genetic vulnerability. Shared heritable vulnerabilities between the two conditions has, however, received little attention in the literature, but may account for some of the overlap between the disorders, and is hence the focus of the present study.

## Overlapping Features Related to the Autism Dyad in Clinical Populations and Their Relatives

### Social Impairment

Evidence for the overlap between AN and autism is related to shared difficulties in important neurocognitive processes related to organisation thoughts and behaviours, and relating to others (Boltri & Sapuppo, 2021). One of these areas is *social cognition* and describes the ability to infer the mental states and perspectives of others, often termed *Theory of Mind* (ToM), or *mentalising* (Baron-Cohen, 2000). As social problems are a key diagnostic feature of autism, difficulties in this domain among autistic populations are well-supported; including studies demonstrating difficulties in identifying and interpreting non-verbal cues such as eye gaze (Wallace et al., 2010), facial expressions (Eack et al., 2014; Fiorentini et al., 2012; Lozier et al., 2014; Sucksmith et al., 2013), and inferring meaning from ambiguous social situations (Baron-Cohen, 2000; Baron-Cohen & Hammer, 1997). It should be noted, however, that difficulty in ToM tasks is not exclusive to autistic populations, nor is ToM difficulty universal among autistic individuals (Gernsbacher, 2019). Task specific nuances, and real-world implications should be considered. While not a diagnostic criterion for AN, a meta-analysis identified that ToM was significantly impaired in individuals with AN, suggesting that difficulties in taking other’s perspectives may be related to perceptual distortions regarding body image and problems with insight common in the disorder (Bora & Köse, 2016). Indeed, Baron-Cohen et al. (2013) explored the presence of traits related to the autism dyad in a sample of adolescent girls and found that those with AN demonstrated significantly lower scores on measures related to empathy, perspective-taking and social awareness, compared to healthy controls. Furthermore, neuroimaging research shows that women with AN present with morphometric (Björnsdotter et al., 2018) and functional (McAdams & Krawczyk, 2011) aberrations in “social brain” regions, akin to those commonly observed in autism (Sato & Uono, 2019).

In support of the heritability theory to explain the overlap in these disorders, difficulties in social relating, and poorer social skills and cognition is also well-documented in parents of autistic children (Baron-Cohen & Hammer, 1997; Bora & Köse, 2016; Rubenstein & Chawla, 2018; Tiede & Walton, 2021). Because these problems are typically less severe in parents and may not necessarily qualify for a diagnosis, they are often conceptualized as part of a *broad autism phenotype* (Wheelwright et al., 2010). Social difficulties in parents have been observed across methodologies and domains; e.g. mothers and fathers of autistic individuals have been shown to have atypical brain activity in response to social stimuli like faces, compared to controls (Dawson et al., 2005; Fiorentini et al., 2012) and exhibit weaker performance on tests designed to assess the ability to recognise emotions (Palermo et al., 2006).

In contrast to autistic populations, research on social cognition deficits among families of individuals with AN is scarce and inconsistent (see Malcolm and Phillipou (2021) for a review). A study from Rhind et al. (2014) did not find evidence of social difficulties relating to the autism dyad among parents of individuals with AN. However, this study relied on self-report questionnaires which may be vulnerable to issues in reflective capacity and insight are hypothesised to be impaired in this group. Conversely, in a study using neurocognitive tasks designed to assess social cognition, Tapajóz et al. (2019) identified that sisters and mothers of individuals with AN performed significantly worse, compared to healthy controls, in identifying correct emotions from ambiguous images.

### Restricted, Repetitive Patterns of Behaviour, Interests, or Activities

A hallmark behavioural characteristic of autism is the presence of RRBI, which can include stereotyped movements, rigid thinking and behaviour, insistence of sameness and obsessive interests in a narrow range of activities (APA, 2022). RRBI are shown to be related to difficulties in the executive functioning domain of *cognitive flexibility*, or *set-shifting*, which refers to the ability to easily adapt behaviour and mental processes to changing contexts (Miller et al., 2015). Given the relationship of cognitive flexibility with RRBI, evidence of impaired cognitive flexibility among autistic individual is well-supported (see Demetriou et al. (2018) for a review). However, cognitive flexibility difficulties have also been observed in several studies among AN populations, manifesting as a preoccupation and fixation with food, dieting and exercise, reminiscent of RRBI in autism (Gillberg, 1985; Oldershaw et al., 2011; Westwood & Tchanturia, 2017). Difficulties in cognitive flexibility have been observed in individuals with AN in both the acute phase of the illness (Tchanturia et al., 2012; Tenconi et al., 2010; Westwood et al., 2016; Zastrow et al., 2009) and also into recovery (Miles et al., 2022; Tenconi et al., 2010; Weinbach et al., 2019). Critically, in AN, those with elevated autistic traits tend to display greater cognitive rigidity (Westwood et al., 2017), reinforcing the potential overlap in underlying neurocognitive mechanisms between autism and AN. This is further supported by neurobiological research. The P3 event related potential (ERP), recorded using electroencephalography (EEG), is a key neural marker of the brain’s reaction when inhibiting a response. This component has been shown to differ in individuals with autism (Høyland et al., 2019) and with feeding and eating disorders (Berchio et al., 2022), compared to neurotypical controls. These differences are particularly evident in response to food-related stimuli (Yan et al., 2023), highlighting the role of atypical neural processing in clinical presentation. Overlap in the neurobiological basis of response inhibition profiles in autism and AN, however, remains unexplored, representing yet another a critical gap in the literature.

From a heritability perspective, while there is a relatively robust body of evidence supporting cognitive flexibility difficulties in parents of autistic individuals (Delorme et al., 2007; Hughes et al., 1997; Rubenstein & Chawla, 2018; Schmitt et al., 2019), the findings are mixed in this domain among parents of children with AN. For example, Weinbach et al. (2019) did not find any differences in cognitive flexibility skills between parents nor siblings of individuals who had recovered from AN, compared to controls. In contrast, Galimberti et al. (2013) found that both individuals with AN and their first-degree relatives performed significantly worse on neuropsychological tasks associated with decision-making and cognitive flexibility, compared to healthy controls. Interestingly, they failed to identify a correlation between illness severity as measured by body mass index (BMI), and performance on these tasks, suggesting that deficits in cognitive flexibility may not be fully accounted for by the neurocognitive impacts of starvation associated with more severe illness. This is supported by research examining executive functioning deficits in family members of individuals with AN (Holliday et al., 2005; Lang et al., 2016) and also in a study among twins, which concluded that impairments in set-shifting could be considered an endophenotype, or a genetic trait that is linked to vulnerability to the disorder (Kanakam et al., 2013).

Thus, difficulties with social cognition and cognitive flexibility could be a function of shared and potentially heritable neurocognitive traits and neurobiological irregularities that present a vulnerability to AN and autism, which might be contributing to the overlap between the two disorders. The literature in this regard, particularly evaluating the relevant neurocognitive profiles of first-degree relatives of those with AN is incredibly limited, highlighting a critical gap and warranting further investigation.

There is a critical gap in the research directly comparing behavioural and neurocognitive profiles in parents of autistic children and parents of children with AN. This is despite growing evidence of an overlap between the two conditions, particularly regarding neurocognitive features related to the autism dyad, including; difficulties in cognitive flexibility (Tchanturia et al., 2016; Westwood et al., 2016) and impaired social cognition (Baron-Cohen et al., 2013; Ghiotto et al., 2022). There is also evidence to suggest that these common traits may be heritable (Delorme et al., 2007; Galimberti et al., 2013; Rubenstein & Chawla, 2018). Increasing understanding of neurocognitive profiles related to the autism dyad in parents of children with either of these disorders can help to better understand risk profiles, and therefore, will allow for early monitoring, improved diagnostic assessment, and the development of targeted interventions.

This study aimed to explore whether there are overlapping behavioural and neurocognitive profiles related to the autism dyad in three groups of parents; parents of children with AN, parents of autistic children, and parents of typically developing children. It was hypothesised that parents of children with a clinical diagnosis of AN, and parents of an autistic child would demonstrate higher traits of eating disorder psychopathology and autism, and also perform worse on neurocognitive assessments compared to parents of typically developing children.

Finally, it was hypothesised that there would be a relationship between eating disorder psychopathology and autistic traits and performance on neurocognitive tasks related to the autism dyad.

## Method

### Participants

A priori power analysis conducted using G*Power revealed that a sample of 159 would be required to detect a medium sized effect, which is consistent with effect sizes from similar studies (Westwood et al., 2016) investigating set-shifting profiles, albeit in clinical (autism and AN) populations, with 0.8 statistical power (α = 0.05). Participants were recruited via online advertisements shared with relevant community support groups (e.g., Autism Awareness Australia, The Butterfly Foundation) and posted on social media (e.g., Twitter, Facebook and Reddit), and research platform Prolific. Ethics approval was obtained from Deakin University Human Research Ethics Committee (DU-HREC, project ID: 2022 − 151).

A total of 324 participants enrolled in the study. Following stringent data cleaning (described below), there were 122 participants who completed the questionnaire component, comprising 41 parents of typically developing children, (age *M =* 37.34 years, *SD =* 8.47), 43 parents of autistic children (age *M =* 41.02 years, *SD =* 9.18) and 38 parents of children with AN (age *M =* 44.03 years, *SD =* 8.30). No significant differences were noted between groups on biological sex or level of education. There were, however, significant differences observed between parent groups on their own self-reported diagnoses, whereby increased psychopathology was present among parents of children in the clinical groups. Refer to Table 1 for participant demographics. A summary of self-reported family history of neurodevelopmental and feeding and eating disorders is provided in Table 2.

**Table 1:** Overview of participant characteristics (Biological sex, education and parent diagnoses)

Demographic Characteristic	Control (*n* = 41)		Parents of autistic children (*n* = 43)		Parents of children with AN (*N* = 38)		χ²	*p*	Cramer’s V
	*n*	%	*n*	%	*n*	%			
Biological sex							4.98	0.083	0.20
Female	21	51.2	19	44.2	26	68.4			
Male	20	48.8	24	55.8	12	31.6			
Education							10.91	0.537	0.21
Did not complete High School	2	4.9	3	7.0	2	5.3			
High school graduate	7	17.1	8	18.6	5	13.2			
Diploma	3	7.3	7	16.3	8	21.1			
Bachelor’s degree	22	53.7	17	39.5	14	36.8			
Masters degree	5	12.2	5	11.6	7	18.4			
Doctoral degree	1	2.4	3	7.0	0	0			
Other	1	2.4	0	0	2	5.3			
Parent Diagnoses							28.66	< 0.001	0.34
None	30	73.2	17	40.5	21	55.3			
Developmental disorder	3	7.3	14	33.3	0	0			
Eating disorder	2	4.9	5	11.9	10	26.3			
Another psychiatric or psychological disorder	6	14.6	6	14.3	7	18.4			

**Table 2:** Summary of self-reported family history of neurodevelopmental or eating disorders

	Control	Parents of Autistic Children	Parents of children with AN
**Neurodevelopmental Disorder**	*n* = 7*	*n* = 4	*n* = 3
Biological Relation	Frequency	Frequency	Frequency
Grandparent		1	1
Parent	1		
Sibling	2	2	1
Aunty/Uncle	2	1	1
Cousin	2		
Niece/Nephew	1		
**Eating Disorder**	*n* = 5	*n* = 4	*n* = 4
Biological Relation	Frequency	Frequency	Frequency
Grandparent			1
Parent			
Sibling	2	2	
Aunty/Uncle		1	1
Cousin	3	1	1
Niece/Nephew			1

Note: * Data for one participant was removed due to reporting of a major medical condition not consistent with a neurodevelopmental disorder. Frequency and sample size data are inconsistent due to one participant noting having multiple biological relatives with diagnoses of neurodevelopmental disorders

Parents in the clinical groups were allocated based on self-report of their child having received assessment and diagnosis by a suitable clinician (e.g., psychologist, psychiatrist, paediatrician). Only parents with children aged 13 and above were eligible for the study, given AN is most commonly diagnosed in adolescence (Smink et al., 2012) and setting an age minimum reduced the possibility of the control group including parents of children who were too young to have received a diagnosis. There were 12 participants who stated that they had a child with both diagnoses. Because there were not enough participants to examine unique differences for this group, these participants were also excluded from the analysis. Likely due to the neurocognitive tasks requiring additional software installation (detailed below), there was significant participant attrition at this point in the online study. Therefore, the data for the neurocognitive tasks comprised a smaller subset of participants (*n* = 68 [control, *n* = 28; Parents of autistic child, *n* = 21; Parents of child with AN, *n* = 19).

## Measures

### Clinical and Behavioural Measures

***The Autism Spectrum Quotient (AQ;*** Baron-Cohen et al., 2001). The AQ is a 50-item self-report questionnaire, measured on a 4-point response scale, designed to assess traits and characteristics relevant to autism in people aged 16 years and above. The AQ was used to characterise traits and characteristics of autism. It comprises five subscales reflecting the core symptoms associated with autism: Social Skills, Attention Switching, Attention to Detail, Communication, and Imagination. The AQ is widely used in both clinical and non-clinical populations, and has good psychometric properties (Baron-Cohen et al., 2001).

***Eating Disorder Examination Questionnaire (EDE-Q;*** Fairburn & Beglin, 1994***).*** The EDE-Q is a 28-item self-report questionnaire which comprises a 7-point rating system and scores to generate data surrounding three sub-scales: restraint, shape concern, and weight concern. This measure is widely used in both clinical and non-clinical populations, and has sound psychometric properties, including excellent internal consistency and test-rest reliability (Luce & Crowther, 1999). A cut-off score of 4.0 is generally considered clinically significant (Carter et al., 2001; Mond et al., 2007).

***The Reflective Functioning Questionnaire (RFQ; (***Fonagy et al., 2016). The RFQ is a self-report measure of intra- and inter-personal mental and emotional social processes, which measures the ability to infer other people’s mental states. The questionnaire comprises 8-items on a 7-point scale (from *Strongly Disagree* to *Strongly Agree*) has reliable psychometric properties (Fonagy et al., 2016).

***The Adult Repetitive Behaviours Questionnaire (RBQ-2 A;*** Barrett et al., 2015). The RBQ-2 A is a 20-item self-report questionnaire assessing RRBI in adults. It includes questions related to adherence to routines, repetitive motor behaviours and sensory interests, and measures their frequency on both a 4-point scale, from *Never or rarely* up to *30 or more times daily*, and a 3-point scale from *Never or rarely* through to *Marked or notable.* This questionnaire is reliable for use in clinical and non-clinical samples (Brett et al., 2023).

### Neurocognitive Tasks

#### Social Cognition

***The Penn Emotion Recognition Test (ER-40;*** Kohler et al., 2003). The ER-40 is a neurocognitive task which involves showing participants 40-colour photographs of human faces, one by one, and asks them to identify the emotion the face represents (including; happy, angry, sad, scared and no feeling). The Research Domain Criteria (RDoC) framework recommends the ER-40 as an appropriate measure to inform Social Communication/Processes (NIMH, n.d).

#### Cognitive Flexibility

***Wisconsin Card Sorting Test (WCST;*** Grant & Berg, 1993***).*** The WCST is a neurocognitive measure of cognitive flexibility and set-shifting, shown to be related to RRBI. This study used the computerised version (Lung & Bertone, 2021). The task involves matching a stimulus card with one of four category cards. The rules for matching the cards are not explicitly communicated to respondents, and patterns instead identified using trial and error. Perseverative errors are identified when a participant fails to adapt their response according to a change in rules. These rules are based on the colour, shape or number of the card. The task is completed over 128-trials.

### Procedure

Upon accessing the study via Qualtrics, participants read the Plain Language Statement and provided consent to participate via checkbox. Following this, participants completed the self-report questionnaires, also in Qualtrics. Throughout the questionnaires, several attention and ‘bot’ checks were administered to identify poor quality responses. Attention checks included questions where only one answer would be realistically possible, for example, participants were asked how often they “Travelled to planet Mars on vacation”, where any response other than 0 days was deemed erroneous. ‘Bot’ checks required participants to provide a text response to describe a displayed image.

Following the questionnaires, participants were directed to the Inquisit platform where they completed the neurocognitive tasks (the WCST and ER-40, presented in random order). These tasks required that participants install Inquisit software to their device and they were provided simple instructions to do so. Following the completion of both the neurocognitive tasks and questionnaires, participants were directed to a separate survey in Qualtrics where they were provided an opportunity to supply their email address and enter a draw to win one of 10 AUD$150 electronic gift cards. The study took participants approximately 45-minutes to complete.

### Data Cleaning and Quality

Several conservative data cleaning checks were undertaken to ensure the quality of the data. Due to the nature of online data collection, it was technically possible that participants could complete the study multiple times. Therefore, considering the degree of ambiguity and uncertainty regarding multiple submissions from the same IP address, all instances of duplicate IP addresses were removed. Participants who failed to provide an adequate response for the ‘bot’ check, or failed two or more attention checks were also excluded. Finally, participants whose date of birth indicated that they did not meet inclusion criteria (aged 18 years or older), were also excluded.

Reliability analysis was conducted for all scales and subscales to understand internal consistency (Supplementary Table S1). Most measures had an α of 0.70 or greater which is considered acceptable (Kline, 2023), with the exception of some AQ subscales which were slightly lower, but consistent with reported reliability results of this measure from previous studies (Hurst et al., 2007).

### Statistical Design

In order to address the first hypotheses to compare clinical profiles and performance on neurocognitive tasks between the three groups of parents, a series of one-way between group Analysis of Variance (ANOVAs) were planned with post-hoc tests calculated where necessary. To account for multiple comparisons, a more conservative α of 0.02 was adopted based on a simple Bonferroni correction. Bayes factors are provided alongside these analyses to aid in interpretation, whereby Bayes factors < 1 are indicative of evidence in support of the null hypothesis (IBM, 2021). We acknowledge that some parent variables might be relevant covariates, an hence additionally ran these analyses with parent biological sex, education and diagnosis included in our model. Relevant statistical assumptions, including assessment of outliers, normality of distribution and residuals, and equality of variance were assessed using protocol described by Tabachnick et al. (2013). Three statistical outliers were identified, using z-scores to indicate values that were three or more standard deviations above or below the mean. These extreme scores were adjusted using a winsorizing approach as described by Field (2013).

Assumptions of normality of distributions and residuals were violated for all but two of the dependent measures (AQ and RBQ total scores), and transformation attempts were unsuccessful. To account for these violations, sensitivity analyses were conducted using equivalent non-parametric tests (Kruskal Wallis) alongside parametric procedures.

To address the final hypothesis, that there would be a significant relationship between autistic traits and eating disorder psychopathology and neurocognitive tasks related to the autism dyad, a series of bivariate correlations were planned. Assumptions of linearity and homoscedasticity were met, yet as mentioned previously, the assumption of normality of was not met for most outcome variables. As correlation analyses are not robust to deviations from normality, a series of Spearman’s rank order correlation were conducted.

## Results

### Differences Between Parent Groups on Behavioural and Clinical Traits

To understand differences in behavioural and clinical traits associated with autism and eating disorder psychopathology, one-way between group ANOVAs were conducted. Results from this analysis examining the self-report data can be found in Table 3. Frequentist analysis revealed a significant group difference on the AQ *Imagination* subscale with a medium effect (η^2^ = 0.07), however this was not supported by the complimentary Bayesian one-way ANOVA, whereby Bayes Factors provided evidence in favour of the null hypothesis for all comparisons. Returning to the frequentist approach, post-hoc tests revealed that parents of autistic children (*M =* 3.62, *SD =* 1.40), endorsed having more difficulties with imagination compared to the control group *(M =* 2.54, *SD =* 1.73), *p* = .01. Imagination sub-scale scores did not differ between parents of children with AN and the control group, nor between the parents of autistic children and parents of children with AN. Of note, when parent’s biological sex, education, and diagnosis were factored into the model, the omnibus test became non-significant (*p* > .05). There were no other significant differences identified between the three groups of parents on these self-report measures, and effect sizes were mostly negligible to small. Sensitivity analysis using the non-parametric Kruskal-Wallace H test lends support to this finding, however, pairwise comparisons did not meet statistical significance, refer to Supplementary Table S2.

**Table 3:** Descriptive and Inferential statistics comparing parent groups on self-reported behavioural and clinical traits associated with autism and AN

Measure	Control (*n* = 41)		Parents of autistic children (*n* = 43)		Parents of children with AN (*n* = 38)		F(2,119)	*p*	η2	Bayes Factor
	M	SD	M	SD	M	SD				
AQ Total	19.76	7.54	22.86	6.38	21.50	8.12	1.88	0.157	0.03	0.05
*Social*	3.66	2.60	3.74	2.36	3.89	2.96	0.08	0.923	0.00	0.01
*Switching*	4.44	2.02	5.33	1.61	4.84	2.21	2.17	0.119	0.04	0.07
*Details*	5.56	2.32	5.65	1.80	5.60	2.30	0.02	0.982	0.00	0.01
*Communication*	3.56	2.64	4.51	2.44	3.89	2.68	1.47	0.235	0.02	0.03
*Imagination*	2.54	1.73	3.63	1.40	3.26	1.91	4.53	0.013*	0.07	0.61
EDE-Q	1.87	1.32	1.81	1.71	1.79	1.31	0.19	0.829	0.00	0.01
*Restraint*	1.85	1.63	1.81	1.71	1.34	1.44	1.24	0.293	0.02	0.03
*Eating*	1.12	1.21	1.55	1.49	1.39	1.34	1.11	0.333	0.02	0.02
*Shape*	2.43	1.64	2.32	1.60	2.31	1.48	0.07	0.930	0.00	0.01
*Weight*	2.10	1.55	2.25	1.74	2.14	1.48	0.09	0.911	0.00	0.01
RBQ	33.39	8.78	38.12	9.80	34.53	9.73	2.87	0.061	0.05	0.13
RFQ Uncertainty	0.55	0.62	0.85	0.83	0.65	0.74	1.86	0.161	0.03	0.05
RFQ Certainty	1.06	0.81	0.96	0.96	0.96	0.94	0.17	0.842	0.00	0.01

Note. *p < .05. AQ = Autism Spectrum Quotient, EDE-Q = Eating Disorder Examination Questionnaire, RBQ = Adult Repetitive Behaviours Questionnaire, RFQ = Reflective Functioning Questionnaire, Bayes Factor = JZS

### Differences Between Parent Groups on Performance on Neurocognitive Tasks

A series of one-way between group ANOVAs were also conducted between the three parent groups on neurocognitive outcomes, refer to Table 4. Parents differed on their total error and perseverative error scores on the WCST, with medium effects. Post-hoc tests revealed that parents of children with AN made significantly fewer total errors on the WCST than parents of autistic children (*M =* 55.33, *SD =* 27.81), *p* = .015. Parents of children with AN also made fewer perseverative errors on the WCST (*M =* 15.37, *SD =* 8.69), compared to the control group (*M =* 29.29, *SD =* 19.68), *p* = .024, and parents of autistic children (*M =* 28.51, *SD =* 19.77) *p* = .051, though these effects do not survive corrections for multiple comparisons. There were no significant differences on the WCST measures between the control group and parents of autistic children. There were also no significant differences between groups on the ER-40. Once again, complimentary Bayesian one-way ANOVA provided evidence in favour of the null hypothesis for all comparisons. Supplementary Table S3 presents complimentary non-parametric Kruskal-Wallace tests complimenting these outcomes. With respect to neurocognitive tasks, again, when parent’s biological sex, education, and diagnosis were factored into the ANOVA model, all result for overall models became non-significant (*p* > .05).

**Table 4:** Descriptive and Inferential statistics comparing parent groups on neurocognitive tasks

Measure	Control (*n* = 28)		Parents of autistic children (*n* = 21)		Parents of children with AN (*n* = 19)		F(2,65)	*p*	η^2^	Bayes Factor
	M	SD	M	SD	M	SD				
WCST										
*Total Errors*	48.61	24.45	55.33	27.81	32.37	23.00	4.38	0.016*	0.12	0.82
*Total Perseverative Errors*	29.29	19.68	28.51	19.77	15.37	8.69	4.19	0.019*	0.11	0.70
ER-40										
*Count Correct*	30.07	8.75	28.57	9.02	29.28	8.69	0.20	0.820	0.01	0.02

Note. *p < .05. WCST = Wisconsin Card Sorting Test, ER-40 = Penn Emotion Recognition Test, Bayes Factor = JZS

### Relationship Between Behavioural and Clinical Traits and Performance on Neurocognitive Tasks

To understand the extent to which neurocognitive profiles were related to behavioural and self-reported clinical traits of autism and eating disorder psychopathology, a series of Spearman’s correlation were conducted (see Table 5). These correlations were in the expected direction and several significant relationships were identified. Due to multiple comparisons being conducted, a simple Bonferroni correction was applied, and α *<* 0.003 was considered to be statistically significant.

**Table 5:** Correlations for study variables

Variable	WCST Number of Perseverative Errors		ER-40 Count Correct	
	Spearman’s rho	*p*	Spearman’s rho	*p*
AQ Total	0.06	0.600	0.01	0.917
*Social*	-0.14	0.238	0.01	0.913
*Switching*	-0.13	0.281	0.13	0.303
*Details*	-0.06	0.620	0.07	0.582
*Communication*	-0.05	0.706	0.03	0.781
*Imagination*	0.23	0.062	-0.22	0.072
EDE-Q	0.21	0.080	-0.35	0.004
*Restraint*	0.31	0.002**	-0.34	0.005
*Eating*	0.25	0.039	-0.40	< 0.001**
*Shape*	0.07	0.575	-0.21	0.094
*Weight*	0.09	0.462	-0.27	0.025
RBQ	0.30	0.015	-0.41	< 0.001**
RFQ Uncertainty	0.21	0.094	-0.06	0.616
RFQ Certainty	-0.04	0.751	0.22	0.078

Note. **statistically significant at *p <* .003 as per Bonferroni adjustment. AQ = Autism Spectrum Quotient, EDE-Q = Eating Disorder Examination Questionnaire, RBQ = Adult Repetitive Behaviours Questionnaire, RFQ = Reflective Functioning Questionnaire, WCST = Wisconsin Card Sorting Test, ER-40 = Penn Emotion Recognition Test

For those relationships that remained statistically significant following correction for multiple comparisons, complimentary analysis were conducted by stratifying the data and running the correlation by parent group. Differences in the strength of these correlations were compared using Fisher’s z, refer to Tables 6 and 7, respectively.

**Table 6:** Correlations with the WCST number of preservative errors across the three groups of parents

Measure	Control (*n* = 28)		Parents of autistic children (*n* = 21)		Parents of children with AN (*n* = 19)	
	*Spearman’s rho*	*p*	*Spearman’s rho*	*p*	*Spearman’s rho*	*p*
*EDE-Q - Restraint*	0.11	0.57	0.37	0.10	0.23	0.21

Note. EDE-Q = Eating Disorder Examination Questionnaire, WCST = Wisconsin Card Sorting Test

**Table 7:** Correlations with the ER-40 count correct across the three groups of parents

Measure	Control (*n* = 28)		Parents of autistic children (*n* = 21)		Parents of children with AN (*n* = 19)	
	*Spearman’s rho*	*p*	*Spearman’s rho*	*p*	*Spearman’s rho*	*p*
EDE-Q- *Eating*	-0.41	0.029	-0.33	0.140	-0.37	0.122
RBQ	-0.21	0.263	-0.77	0.001**	-0.29	0.233

Note. **statistically significant at *p <* .003 as per Bonferroni adjustment. EDE-Q = Eating Disorder Examination Questionnaire, ER-40 = Penn Emotion Recognition Test

Within groups, no significant relationships (*p* = > 0.05) between the EDE-Q restraint subscale and the number of perseverative errors on the WCST were identified.

A significant negative relationship was identified between RBQ scores and correct responses on the ER-40 for parents of autistic children, but not the other groups. This was significantly stronger than the relationship observed between these variables among parents of children with AN (*z* = -2.10, *p* = .02) and that of parents of typically developing children (*z* = -2.61, *p* = .005). A trend level relationship was also observed between the EDE-Q-eating subscale and correct responses on the ER-40 among parents of typically developing children, though this would not survive correction for multiple comparisons.

## Discussion

There is growing evidence to suggest an overlap between autism and AN (Huke et al., 2013; Nickel et al., 2019; Oldershaw et al., 2011; Saure et al., 2020; Susanin et al., 2022; Westwood & Tchanturia, 2017), both of which are known to be highly heritable (Bulik et al., 2007; Tick et al., 2016). The present study provides a preliminary exploration of the overlapping behavioural and clinical characteristics and neurocognitive profiles related to the autism dyad among parents of children with either disorder, compared to parents of children without such diagnoses. It was firstly hypothesised that parents of children with AN, and parents of autistic children would demonstrate higher traits of AN and autism compared to parents of children with no history of neurodevelopmental or eating disorders. This hypothesis was partially supported; there was a significant difference identified in imaginative abilities among parents of autistic children, compared to the other two parent groups. There were no other significant differences on levels of autistic traits or eating disorder psychopathology between groups. It was also hypothesised that parents of children with AN and parents of autistic children would exhibit weaker performance on neurocognitive tasks assessing cognitive flexibility and social cognition compared to parents of typically developing children. This hypothesis was not supported when examining social cognition, as there were no significant differences identified in performance on the emotional recognition task between the three parent groups. Regarding measures of cognitive flexibility, evidence relevant to this hypothesis was contrary to expectation. It was identified that parents of children with AN had significantly higher performance on the cognitive flexibility task, compared to parents of autistic children and parents of typically developing children. This result was unexpected and converse to the anticipated direction of the relationship. Finally, it was hypothesised that there would be a significant relationship between eating disorder psychopathology and autistic traits and performance on neurocognitive tasks related to the autism dyad. This hypothesis was partially supported, as there was evidence of a significant relationship between performance in both the emotion recognition and cognitive flexibility tasks with eating disorder psychopathology. Specifically, higher scores in some domains of eating disorder psychopathology were associated with making more perseverative errors on tests of cognitive flexibility, and poorer performance on the emotion recognition tasks. Similarly, it was also identified that higher scores on self-reported restricted and repetitive behaviours would be associated with more perseverative errors on cognitive flexibility tasks. The strength of these relationships, however, varied between parent groups. The implications of these findings and position in the current literature are discussed herein.

### Social Cognition

Difficulties in the domain of face/emotion processing are related to deficits in social communication that form part of the dyad of criteria for diagnosing autism. Accordingly, difficulties in social cognition broadly among individuals with autism are well documented (Baron-Cohen, 2000; Demetriou et al., 2018; Sucksmith et al., 2013; Wallace et al., 2010). There is emerging evidence of differences in this domain among individuals with AN, with researchers suggesting that social cognition difficulties could be related to distortions in body in image and difficulties relating to others (Baron-Cohen et al., 2013; Bora & Köse, 2016), indicating evidence for the overlap between the two disorders. The correlational findings in this study are consistent with previous literature (Baron-Cohen et al., 2013; Boltri & Sapuppo, 2021; Bora & Köse, 2016) and support the association between increased eating disorder psychopathology, and deficits in the social aspect of the autism dyad. This relationship was noted at a total sample level, irrespective of the respondent having a child with AN, or ASD, or not. This suggests that the association between AN symptomology and social cognition difficulties may be observable at a general population level. Indeed, this finding supports research from Fithall et al. (2023), which found a significant relationship between eating disorder psychopathology, and difficulties in self-reported mentalising ability among a broad sample of young adults. A finding consistent with similar research in non-clinical samples (Christensen et al., 2019).

The literature with respect to the (social) neurocognitive profiles of parents of autistic children is inconsistent. Several previous studies have identified social cognition difficulties among *parents* of autistic children (Rubenstein & Chawla, 2018; Tiede & Walton, 2021), while others have not (Hill et al., 2004). Stimulus delivery might account for some of this variability, as one study only found difficulties among parents of autistic children in voice-based emotional recognition tasks as opposed to visual (Tajmirriyahi et al., 2013). There is scarce evidence of this same difficulty among parents of children with AN (Malcolm & Phillipou, 2021). There could be several reasons for this inconsistency, including the heterogeneity that exists generally in both the presentation of social communication symptoms and its manifestation in undiagnosed relatives (Bora & Köse, 2016). Social cognition itself is a broad concept, and underlying processes of difficulties in social symptoms related to the autism dyad are complex. Some researchers report that social difficulties are due to problems processing emotional information (Hill et al., 2004) while others assert the difficulties involve representing social and emotional information in the mind (Baron-Cohen, 2000). This is an important distinction of the context of the findings in the present study, that parents of autistic children reported increased difficulties on imaginative abilities. Some researchers suggest that imagination, and the ability to create abstract representations in the mind are an important element of processing social information (Ten Eycke & Müller, 2015). Imagination is also an important aspect of creativity, and difficulties in this domain are consistent with findings from Wong et al. (2006), who identified that parents of autistic children had more difficulties in *idea generation* compared to healthy controls. While impairments in imaginative abilities may be suggestive of a heritable feature in autism, it does not necessarily account for the overlap with AN. Hence, while the current study found an association between eating disorder psychopathology and aspects of social cognition, the heritability aspect of this domain among families of children with AN remains unclear.

### Cognitive Flexibility

This study demonstrated a link between poorer performance on measures of cognitive flexibility and two important domains; eating disorder psychopathology, and self-reported restricted and repetitive behaviours. The presence of difficulties with cognitive flexibility is consistent with literature investigating executive functioning among individuals with AN (Tchanturia et al., 2012; Zastrow et al., 2009). The link between flexible thinking and rigid and restricted behaviours is also consistent with prior research (Demetriou et al., 2018) and adds evidence to the idea that this neurocognitive difficulty may be a shared feature among people with autism and AN. However, while prior research has suggested a heritable link with autism (Schmitt et al., 2019), this effect was not observed in the present study. Unexpectedly, it was found that parents of children with AN had significantly better performance on cognitive flexibility tasks compared to both controls and parents of children with ASD. This finding is contrary to research among first-degree relatives of those with AN which have identified deficits rather than strengths in this area (Galimberti et al., 2013; Holliday et al., 2005). However, superior cognitive flexibility among AN populations is not a completely novel finding. In a study among adolescents who had recovered from AN, Weinbach et al. (2019) observed superior verbal and visual set-shifting abilities compared to healthy controls. The authors attributed this strength to enhanced *inhibitory control*, that is, the capacity to selectively control one’s thoughts and impulses. A later study by the same group indicated that this effect occurs also when presented with disorder-salient (food)s stimuli (Weinbach et al., 2020). To our knowledge, this study is the first to show this similar behavioural pattern among parents of children with AN.

### Implications for Understanding Heritability of the Autism Dyad and Overlap with AN

This study had diverse findings, and while there were clear associations between cognitive flexibility and social cognition difficulties and self-reported AN and autistic traits, differences were not uniformly observed across the parent groups. This finding highlights the complexity in identifying heritable factors in these conditions more generally. Considerable research has attempted to identify unique genetic traits associated with autism and AN (Bulik et al., 2007; Christiansen et al., 2024; Gaugler et al., 2014; Thapar & Rutter, 2021). Difficulties exist in identifying these heritable factors due to considerations regarding the role the environment plays in the manifestation of symptoms (Thapar & Rutter, 2021), and that some genetic variations implicated in autism are also found in undiagnosed populations (Robinson et al., 2016). Therefore, it is possible that there is an underlying heritable component to overlapping neurocognitive traits, but that these are influenced by environmental factors, so that patterns may not be distinguishable by comparing groups of parents. For example, research from Keys et al. (1950) found that nutritional deficiencies can impair executive functioning, and some suggest these changes are reminiscent of symptoms of AN (van Noort et al., 2016). While the correlational findings in the present study support the notion that increased eating disorder psychopathology is associated with more autistic traits, because this is a predominantly non-clinical sample, it suggests that the relationship is unlikely the result of acute effects of starvation in isolation. Indeed, despite some reports of a history of eating disorders among the parent groups, only nine participants of the total sample scored above the clinical-cut off for the EDE-Q, and these participants were distributed across the three parent groups (control, *n* = 3; autism, *n* = 4; AN, *n* = 2), so it is unlikely that their individual scores had a great effect on the results. This, therefore, may lend support to the idea that the observed differences are due to the other factors, including an underlying heritable component.

### Implications for Diagnosis and Treatment

Despite mixed evidence for a heritable vulnerability, the correlational evidence in this study demonstrates a relationship between eating disorder psychopathology and aspects of the autism dyad. While preliminary and exploratory in nature, this association adds to the body of literature exploring an overlap between the disorders, and provides some evidence to account for their comorbidity as well as the misdiagnoses that may occur among autistic individuals, particularly among females (Kirkovski et al., 2013). For example, it is possible that an individual with inherent difficulties in social cognition and cognitive flexibility present with symptoms consistent with AN, such as a fixed focus on food and dieting, leading to misdiagnoses (Brown & Stokes, 2020). Furthermore, in this study, we found that parents who endorsed more behaviours related to eating disorder psychopathology had more problems on neurocognitive tasks related to the autism dyad. It is important to note however, that overall mean scores for the EDE-Q in this sample did not reach clinical cut-offs (i.e. 4) in any of the three groups, demonstrating that the relationship between these features may exist at a subclinical level. Consequently, early difficulties in understanding emotions of others and in executive function could indicate increased risk for future problems related to restricted eating and risk of developing an eating disorder. Regarding treatment, there is promising evidence for adjunct treatments for individuals with AN including cognitive remediation therapy (CRT) (Tchanturia et al., 2014). These treatments involve addressing neuropsychological difficulties associated with AN such as cognitive flexibility and ‘big picture’ thinking, and show promise among AN populations with higher autistic traits (Tchanturia et al., 2016). By identifying a relationship between subclinical eating disorder symptomology and difficulties in neurocognitive features, this study provides further support for the inclusion of adjunct social cognition and cognitive flexibility interventions, particularly for early intervention. This is particularly important given the high increased risk of adverse health outcomes if AN is untreated (Cass et al., 2020) and the high mortality rate for the disorder (Hoek, 2006).

### Limitations and Future Directions

Despite the strengths of this study, there are several limitations that should be noted. The preliminary research presented here is highly exploratory, and should be interpreted with caution. Methodologically, our power calculations were conducted considering effect sizes observed between clinical (autism and AN) and control groups. A number of our participants had not received such a diagnosis themselves, and might have presented with only sub-clinical characteristics. Our study is likely underpowered to detect the potentially smaller effects of the present sample. Furthermore, our initial target sample size of 159 participants was not able to be met (*n* = 122; clinical and behavioural measures), thus further contributing to the likelihood of being underpowered, particularly regarding the neurocognitive assessments (*n* = 68). This is further complicated by the dearth of available literature, making it difficult to estimate expected effect sizes. Second, due to the online approach to data collection, there was a reliance on parents self-reporting information regarding their child’s diagnosis, and this was not confirmed via clinical assessment by the research team. Consequently, the severity of the child’s condition/s could not be considered here. Further with regard to the online self-report nature of this study, the conditions under which the study was completed could not be evaluated, which might impact upon response accuracy. Future research in this field should evaluate the clinical, behavioural, and neurocognitive profiles of parents and children who are impacted by these conditions in a face-to-face format, to mitigate these confounds. Coupled with our conservative approach to data collection, given recruitment channels were carefully selected for this study, and advertisements targeted toward community support groups for autism and AN, it is unlikely this was a major confound. To further extend on our knowledge regarding the heritable and overlapping nature of these conditions, future research could also consider more thorough assessment of relevant family histories more broadly.

A further limitation relates to the role of biological sex of the participating parent and the potential impact on heritability. Specifically, there is evidence to suggest that vulnerability to autism is inherited paternally. Indeed, some researchers have identified more marked deficits in neurocognitive traits and the presence of autism symptoms among fathers rather than mothers (Palermo et al., 2006; Szatmari et al., 2008). Similarly, much research on heritability of AN has focused on heritability through female relatives (Galimberti et al., 2013; Holliday et al., 2005; Lang et al., 2016). The present study did not have adequate sample size to investigate the impact of parent’s biological sex in relation to autism and AN psychopathology, or on performance on neurocognitive tasks. However, analysis was conducted to ensure an equivalent representation of mothers and fathers across the three groups, to minimise any systematic patterns that may have impacted the findings. Nevertheless, there is an opportunity for future research to explore heritability of the autism dyad according to the biological sex of the parent.

Another factor to consider is that a number of parents reported having psychiatric or psychological diagnoses other than eating or neurodevelopmental conditions. It is, therefore, important to consider the extent to which the observed effects are related to phenotypes associated with autism or AN, versus other conditions not investigated here.

Finally, it should also be considered that there are several other neurocognitive domains that were not explored in the present study that could further elucidate the overlap between AN and autism. These include; inhibitory control (Norton et al., 2024; Tonizzi et al., 2022; Weinbach et al., 2019; Wierenga et al., 2014) and central coherence (Kanakam et al., 2013), warranting further research.

## Conclusion

Given the above-described limitations and exploratory nature of this research, it is imperative that any interpretations be made with caution. Notwithstanding, the results of this study do provide additional support to the growing evidence of an overlap between autism and AN as cited in previous literature (Baron-Cohen et al., 2013; Boltri & Sapuppo, 2021; Brown & Stokes, 2020; Fithall et al., 2023; Gillberg, 1985; Huke et al., 2013; Oldershaw et al., 2011; Susanin et al., 2022; Tchanturia et al., 2013; Westwood & Tchanturia, 2017). Specifically, our research demonstrated a significant association between eating disorder pathology and neurocognitive traits associated with the autism dyad, specifically, social cognition and cognitive flexibility. Although this study did not provide evidence of specific or detectable deficits in these domains among the parent groups, these preliminary findings do reveal promising avenues for future research into heritable factors for autism and for AN. .

## Electronic supplementary material

Below is the link to the electronic supplementary material.

Supplementary Material 1

## Data Availability

The datasets used and/or analysed during the current study are available from the corresponding author on reasonable request.
