# Supplementary material for: Neurocognitive Profiles in Parents of Autistic Children and Parents of Children with Anorexia Nervosa
Source: J Autism Dev Disord. 2025 May 15;56(11):4550–65. doi: 10.1007/s10803-025-06871-3 (PMC13687177; doi:10.1007/s10803-025-06871-3)
Supplement: Supplementary file 1 [file 10803_2025_6871_MOESM1_ESM.docx]

**Neurocognitive profiles in parents of autistic children and parents of children with anorexia nervosa**

Supplementary Material

**Supplementary Table S1**

*Internal Reliability Analysis of Questionnaire scales*

|  | Cronbach’s α |
| --- | --- |
| AQ | 0.89 |
| Social | 0.77 |
| Switching | 0.64 |
| Detail | 0.67 |
| Communication | 0.76 |
| Imagination | 0.65 |
| EDE-Q | 0.90 |
| Restraint | 0.88 |
| Eating | 0.84 |
| Shape | 0.89 |
| Weight | 0.82 |
| RFQ | 0.86 |
| Certainty | 0.86 |
| Uncertainty | 0.79 |
| RBQ | 0.93 |

*Note.* AQ = Autism Spectrum Quotient, EDE-Q = Eating Disorder Examination Questionnaire, RBQ = Adult Repetitive Behaviours Questionnaire, RFQ = Reflective Functioning Questionnaire.

**Supplementary Table S2**

*Non-parametric between-group comparisons for self-report questionnaires (Kruskal Wallis Test)*

| *Measure* | *H(2,119)* | | *p* |
| --- | --- | --- | --- |
|  |  |  |  |
| AQ Total | 3.54 | .170 | |
| *Social* | 0.25 | .881 | |
| *Switching* | 4.77 | .920 | |
| *Details* | 0.23 | .890 | |
| *Communication* | 2.87 | .238 | |
| *Imagination* | 9.96 | .007* | |
| EDE-Q | 0.11 | .949 | |
| *Restraint* | 2.57 | .276 | |
| *Eating* | 1.33 | .515 | |
| *Shape* | 0.12 | .944 | |
| *Weight* | 0.11 | .945 | |
| RBQ | 5.73 | .057 | |
| RFQ Uncertainty | 3.93 | .140 | |
| RFQ Certainty | 1.09 | .581 | |

*Note.* **p* < .05. AQ = Autism Spectrum Quotient, EDE-Q = Eating Disorder Examination Questionnaire, RBQ = Adult Repetitive Behaviours Questionnaire, RFQ = Reflective Functioning Questionnaire.

**Supplementary Table S3**

*Non-parametric between group comparisons for neurocognitive tasks (Kruskal Wallis)*

| *Measure* | *H(2,65)* | | *p* |
| --- | --- | --- | --- |
|  |  |  |  |
| WCST |  |  | |
| *Count Errors* | 8.68 | .013* | |
| *% Errors* | 8.23 | .016* | |
| *Count Perseverative Errors* | 8.91 | .012* | |
| *% Perseverative errors* | 7.33 | .026* | |
| ER-40 |  |  | |
| *Count Correct* | 1.59 | .450 | |
| *% Correct* | 1.59 | .450 | |

*Note.* **p* < .05. WCST = Wisconsin Card Sorting Test, ER-40 = Penn Emotion Recognition Test.
